# Cerebral Haemodynamic Changes in Type 2 Diabetes Mellitus Following a Three-Month Yoga Intervention: A Randomized Controlled Trial

**DOI:** 10.7759/cureus.51548

**Published:** 2024-01-02

**Authors:** Mahadevappa Vidyashree, Singh Deepeshwar, Manjunath N K, Chidananda Kaligal, Amit Kanthi, Dwivedi Krishna, Nagarathna Raghuram, Lokesh Bathala, Vijay K Sharma

**Affiliations:** 1 Department of Yoga and Life Sciences, Swami Vivekananda Yoga Anusandhana Samsthana, Deemed to be University, Bangalore, IND; 2 Department of Yoga and Life Sciences, Swami Vivekananda Yoga Anusandhana Samsthana, Bengaluru, IND; 3 Department of Yoga, Babasaheb Bhimrao Ambedkar University, Lucknow, IND; 4 Department of Yoga and Life Sciences, Swami Vivekananda Yoga Anusandhana Samsthana, Bangalore, IND; 5 Department of Neurology, Aster CMI, Bangalore, IND; 6 Department of Neurology, Yong Loo Lin School of Medicine, Singapore, SGP

**Keywords:** yoga research, yoga therapy, corsi block tapping test, working memory, cerebro vascular reactivity, cerebral haemodynamics, transcranial doppler sonography, types 2 diabetes

## Abstract

Background and purpose

Cerebral haemodynamics and cognitive performance may be adversely affected in type 2 diabetes mellitus (T2DM). Previous studies reported reduced cerebral blood flow (CBF) and altered cerebrovascular reactivity (CVR) in T2DM. Yoga, an ancient holistic health approach, is known to be beneficial for T2DM. We hypothesized that yoga practice may alter CBF and the flow resistance in the middle cerebral artery (MCA) and improve cognition in T2DM. Our secondary objective was to explore the relationship between changes in cerebral haemodynamics and cognition in T2DM.

Materials and methods

Participants were randomly allotted into the yoga and control groups based on the eligibility criteria. One hour of yoga intervention specific to type 2 diabetes was provided to the yoga group for three months, while conventional treatment was provided to the control group. A transcranial Doppler was used to evaluate longitudinal changes in cerebral haemodynamics in MCA. A Corsi block tapping test was used to assess visio-spatial working memory.

Results

There were 75 participants recruited, of whom 38 participated in yoga and 37 participated in a control group. Both intention to treat and per protocol analysis showed significant results. At day 90, intention-to-treat analysis showed significant changes in CBF velocities (mean difference −10.85%, 95% CI (−13.26, −6.15), p<0.001), cerebral vasodilatory reserve (mean difference −0.23%, 95% CI (−0.43, -0.03), p=0.02) and cognition (mean difference −12.13%, 95% CI (−17.48, -6.78), p≤0.001). There was no between-group effect. Also, the correlation between the CBF and cognition did not show any significant results.

Conclusion

The three-month yoga intervention was associated with an improvement in cerebral hemodynamics. The study also revealed an improvement in visio-spatial working memory among patients with T2DM. The study did not show any correlation between the improvement in cerebral haemodynamics and working memory. We recommend larger and longer studies on yoga intervention for T2DM patients to evaluate whether such benefits are sustained and improve their quality of life.

## Introduction

Type 2 diabetes mellitus (T2DM) is known to impair cerebral haemodynamics and cognitive performance [1] adversely. Of the total cardiac output, about 20% of blood flow goes to the brain and cerebral blood flow (CBF) is constantly maintained by the homeostatic process of cerebral autoregulation (CA) [2]. Previous studies have reported reduced CBF in patients with T2DM when compared to the healthy population [3]. Furthermore, cerebrovascular reactivity (CVR) may be compromised in T2DM patients [4]. Among patients with T2DM, impaired cerebral haemodynamics is directly related to the duration of the disease, old age, and associated comorbid conditions like hypertension [5]. Mechanisms responsible for the altered cerebral haemodynamics include endothelial and vascular smooth muscle dysfunctions, which in turn contribute towards atherosclerosis [6]. With the increasing prevalence of the disease, regular screening and health education are essential to reduce the risk of complications from T2DM [7].

The middle cerebral artery (MCA) is the most common intracranial artery that is pathologically affected in T2DM patients through embolization, lenticulostriate infarctions, and saccular aneurysms [8]. Additionally, atherosclerotic stenosis of the MCA is known to be associated with white matter changes leading to cognitive impairment, depression, and gait impairment [9]. Cognitive functional decline due to MCA stenosis may affect attention, executive function, and information processing speed [10].

Yoga, an ancient holistic health approach, has been described as beneficial to patients with type 2 diabetes mellitus. Studies have shown that yoga reduces fasting (FBS) and postprandial (PPBS) blood glucose as well as glycated haemoglobin (HbA1c) [11]. In addition, it improves insulin sensitivity and regulates diabetes-related complications such as dyslipidaemia by reducing low-density lipids, cholesterol, and triglycerides [12,13].

Yoga is also known to influence cerebral haemodynamics [14]. Studies have shown that yogic techniques increase CBF in the dorsal medial frontal lobe, prefrontal cortex, and sensorimotor cortex [15]. While breathing practices during ‘bhastrika’ (bellow breathing) reduce the CBF, CBF increases during kumbhaka (inhale and hold) [16]. However, such studies were performed on healthy human beings. To the best of our knowledge, the impact of yoga interventions on cerebral haemodynamic and cognition in patients with T2DM has never been described. Therefore, we aimed to assess the effect of yoga on cerebral haemodynamics and its relation to cognitive performance in patients with T2DM. We hypothesized that yoga practice may alter CBF and the flow resistance in the MCA and improve cognition in T2DM. Our secondary objective was to explore the relationship between changes in cerebral haemodynamics and cognition in T2DM.

## Materials and methods

Participants

We recruited participants for our study through word-of-mouth in our institution, social media, and advertisements placed in various residential localities and health centres in Bengaluru, India, between 2018 and 2021. Participants diagnosed with T2DM based on the guidelines of the American Diabetes Association (2018) [17], who had no incidence of hypoglycaemia for the past two years, were not practicing yoga, had a Montreal cognitive assessment score (MOCA) > 26 points and could give written consent to participate in this study, were recruited.

Patients with obesity (body mass index >30 kg/M2), diabetic neuropathy, diabetic nephropathy, diabetic retinopathy, any major surgical intervention within six months, the presence of any behavioural/psychological conditions screened using checklist-90-R (SCL-90-R) [18], the intake of medication known to influence cognition (corticosteroids, anticonvulsants, and anticholinergic drugs), a history of alcohol or drug abuse within six months and cerebrovascular diseases (transient ischemic attack and stroke) were excluded from the study.

Standard protocol approval

The study methodology was approved by the institutional ethical committee/institutional review board, Swami Vivekananda Yoga Anusandhana Samsthana, deemed to be a university in Bangalore (RES/IEC-SVYASA/109/2017). The study was registered in the Clinical Trials Registry of India (CTRI No. CTRI/2017/12/010936). All the participants provided written informed consent to participate in the study.

Study design

Subjects were randomly assigned to the yoga group or the control group (standard medical therapy) based on random number generation. The data were collected at the beginning of the intervention, at 45±7 days, and finally at 90±7 days.

Yoga intervention

In addition to their standard medications, participants allocated to the yoga intervention group received a T2DM-specific yoga module every three days for three months from a qualified yoga instructor. In the remaining days, they were asked to practice the same module at home. The yoga module was in line with ADA lifestyle recommendations. Yoga practices included loosening exercises, breathing exercises, asanas (physical postures), surya namaskara (sun salutation), pranayama (yogic breathing techniques), and relaxation techniques. Yoga practices are listed in Table 1.

**Table 1:** Yoga Intervention Yoga protocol practiced by the intervention group.

Sl no	Name of the practice	Rounds	Duration
1	Starting prayer		2 min
2	Preparatory practices		
	Sukshma and shitilikarana vyayama (loosening practices)	1 round	4 min
	Hand stretch breathing (90 ^◦^C, 145 ^◦^C, and 180 ^◦^C)	3 rounds	2 min
	Hands in and out breathing	3 rounds	1 min
	Forward and backward bending	3 rounds	1 min
	Twisting	3 rounds	1 min
	Sarvanga Pushti (clockwise and anti-clockwise)	3 rounds	1 min
3	Surya namaskar (sun salutation)		
	10 steps fast Surya namaskar (sun salutation)	6 rounds	8 min
	12 steps slow Surya namaskar (sun salutation)	1 round	2 min
4	Asana (postures)		
	Standing: Ardhakaticakrasana (lateral arc pose), ardha cakrasana (half wheel pose), Trikonasana (triangular pose), Parivritta trikonasana (revolved triangular pose), Prasarita padahastansana (wide stance forward bend pose), vriksana (tree pose)	1 round each	5 min
	Sitting: Mandukasana (frog pose), Vakrasana/Ardamathsyendrasana (twisted pose), Paschimotanasana (seated forward bend pose), Ardha Ustrasana (half camel pose)	1 round each	5 min
	Prone: Bhujangasana (serpent pose), Dhanurasana (bow pose)	1 round each	5 min
	Supine: Jatara parivartanasana (spinal twist pose), Pavanamuktasana (wind relieving pose), Viparitakarani (legs up the wall pose), Navasana (boat pose), Relaxation in savasana with deep abdominal breathing.	1 round each	5 min
5	Kriya		
	Agnisara (rigorous movement of the abdomen)	1 round	2 min
	Kapalabhati (forceful exhalation)	2 round	2 min
6	Pranayama		
	Nadishuddi (alternate nostril breathing)	9 rounds each	3 min
	Bhramari (female humming bee sound)	9 rounds each	3 min
7	Resolve (I am completely healthy)		2 min
8	Closing prayer		2 min
			Total- 60 min

Control group

The control group received general guidelines for maintaining blood glucose levels. They continued their routine along with their conventional treatment. They were given awareness regarding the diet modalities in DM. The study monitored any changes in medication dosages.

Assessments

Cerebral Haemodynamics Assessments

The cerebral haemodynamics were assessed by a single operator (MV) using transcranial Doppler (TCD) (Multi Dop X, DWL Compumedics, Germany). A 2-MHz TCD ultrasound transducer probe was placed in the temporal area above the zygomatic arch and in front of the tragus of the ear. The probe was adjusted to get a consistent spectral flow of MCA between the depths of 45 and 60 mm and recorded peak systolic velocity (PSV), end-diastolic velocity (EDV), mean flow velocity (MFV), pulsatility index (PI), resistance index (RI) and CVR using the breath-holding test. During the breath-holding test, baseline reading was done for two minutes, followed by asking the subjects to hold their breath for 30 seconds or as long as they could hold comfortably. CVR was assessed using the breath-holding index (BHI), which is calculated as the percentage of velocity increase after breath-holding from the resting baseline values and divided by the breath-holding time [19].

Cognitive Assessment

A computerized version of the Corsi block tapping test (both forward and backward) was administered to the subjects using Inquisit software to measure their visuospatial working memory. In this test, the computer screen displayed nine squares of the same colour with a black background. Squares started flashing in a sequence and subjects remembered the same sequence for the forward Corsi block tapping test and its reverse order for the backward Corsi block tapping test. They responded by clicking the mouse on the desired squares [20].

Statistical analysis

Data were analyzed with SPSS statistics (IBM SPSS Statistics for Windows, Version 23.0, IBM Corp., Armonk, NY). The data were tested for normality using the Shapiro-Wilk test. An RM-ANOVA was conducted to analyze the statistical changes within and between groups for all the parameters separately. A post-hoc pairwise comparison with a Bonferroni correction was done. Both intention-to-treat (ITT) analysis and per-protocol (PPA) analysis were conducted. The missing value analysis was done before the ITT analysis. Pearson’s correlation test was followed to check the association between CBF and cognition.

## Results

**Figure 1:**
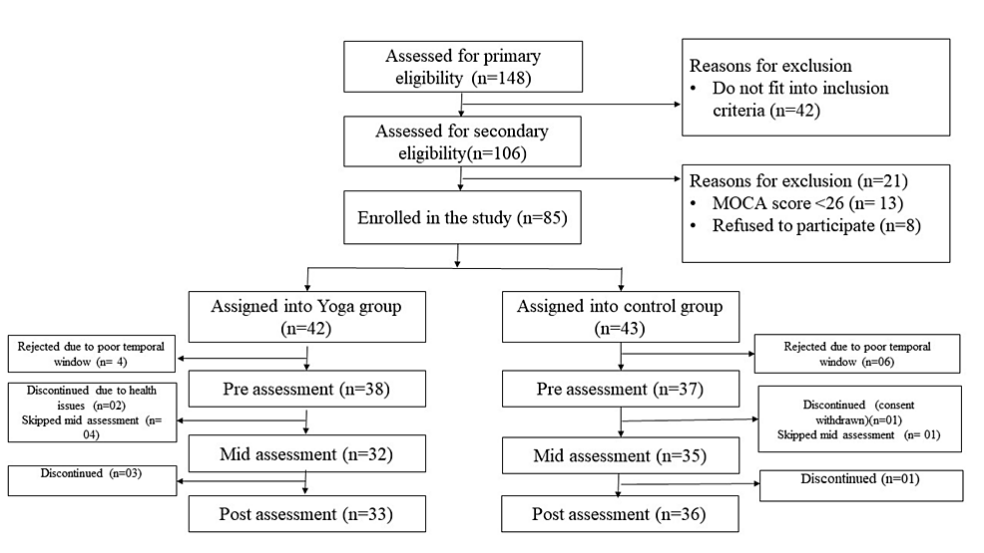
CONSORT flow chart CONSORT flow chart showcasing the number of participants recruited and assessed during the study period.

The study recruited a total of 75 participants, with a mean (SD) age of 51.5± 9.8 years and 63 (84%) men. There were 38 subjects in the yoga intervention group and 37 subjects in the control group. Figure 1 depicts the CONSORT flow chart for the subject’s participation in the study.

The two groups were well matched for age (yoga intervention group 52.9±8.2 years and 49.6±10.9 years in the control group) and MOCA score (yoga intervention group 27.1±1.1 and 27.1±0.9 in the control group). Table 2 presents the demographic details of the subjects.

**Table 2:** Demographic details of the yoga group and control group INR: Indian Rupees; MOCA: Montreal cognitive assessment score.

	Yoga intervention group (n-38)	Control group (n-37)
Age in years (mean± SD)	52.9±8.2	49.6±10.9
Gender, n (%)		
Female	7 (18.4 %)	6 (16.2%)
Male	31 (81.6 %)	31 (83.8%)
Duration of T2DM in years (Mean ± SD)	8.2±6.4	7.4±4.5
Hypertension, n (%)	12 (31.6%)	14 (37.8%)
Mean MOCA Score ± SD	27.13±1.10	27.11±0.95
Marital status, n (%)		
Married	38 (100%)	35(94.5%)
Single	0	2 (5.5%)
Education, n (%)		
Primary school	0	1 (2.7%)
High school	2 (5.3%)	3 (8.1%)
Graduation	27 (71.1%)	26 (68.4%)
Post-graduation	9 (23.7%)	7 (18.9%)
Annual income in INR, n (%)		
<2 lakh	6 (15.8%)	7 (18.4%)
2–5 lakh	10 (26.3%)	5 (13.2%)
5–10 lakh	10 (26.3%)	3 (7.9%)
>10 lakh	12 (31.6%)	22 (57.9%)
Food habit, n (%)		
Vegetarian	31 (81.6%)	27 (71.1%)
Non-vegetarian	7 (18.4%)	10 (26.3%)
Employment, n (%)		
Full time	22 (57.9%)	26 (68.4%)
Retired	11 (28.9%)	7 (18.4%)
Not working	5 (13.2%)	4 (10.5%)

Table 3 shows the longitudinal changes in various physical and biochemical parameters in both study groups. Briefly, by day 90, significant reductions were noted in the body mass index (2.14%), waist circumference (0.68%), and hip circumference (0.73%) among subjects in the yoga intervention group. Systolic (4.9%) and diastolic blood pressure (6.8%) also showed significant reductions at the end of the study in the yoga intervention group. Furthermore, diabetic biochemical parameters also showed significant reductions in the yoga intervention group (fasting blood glucose reduced by 9.4%, post-prandial blood glucose reduced by 16.09%, and glycated haemoglobin by 6.7%) at three months. There were no changes in the medication score in the yoga group.

**Table 3:** Descriptive statistics of yoga and control group BMI: body mass index, BP: blood pressure, FBS: fasting blood sugar, OHD: oral hypoglycaemic drugs, PPBS: post-prandial blood sugar.

Group	Yoga Group					Control Group				
Parameters	Baseline (mean ± SD)	Day-45 (mean ± SD)	Day-90 (mean ± SD)	P-value at day-90	Cohen’s d	Baseline (mean ± SD)	Day-45 (mean ± SD)	Day-90 (mean ± SD)	P-value at day-90	Cohen’s d
Mean BMI ± SD (kg/m^2^)	25.7±3.2	25.5±3.2	25.15±3.2	<0.00	0.17	26.8±4.2	27.2 ±4.6	27.16±4.54	0.29	0.08
Waist circumference (cm)	94.58±7.74	94.01±7.21	93.93 ± 18.3	0.032	0.04	92.3±6.7	91.63±6.79	91.79±6.40	0.49	0.07
Hip circumference (cm)	98.62±6.9	98.21±6.9	97.9±6.9	0.009	0.10	95.04±7.82	95.55±9.29	95.06±9.07	0.84	0.002
Mean systolic BP±SD (mm of Hg)	119.16±10.7	114.6± 21.5	113.3±5.6	0.001	0.68	123.6±11.7	120.9±10.81	120.65±12.97	0.21	0.23
Mean diastolic BP±SD (mm of Hg)	76.16±7.97	72.44±6.8	71.00±7.22	0.001	0.67	78.1± 7.8	76.49±7.8	75.65±8.7	0.08	0.29
Mean pulse rate± SD (bpm)	79.39±6.56	78.89±7.8	76.2±9.42	0.039	0.39	81.16±7.28	80.98±7.52	79.71±6.35	0.14	0.21
Mean FBS ± SD (mg/dl)	148.82±41.27	148.74±36.5	134.82±30.46	0.006	0.38	136.81±33.5	137.25±33.76	129.59±28.09	0.62	0.23
Mean PPBS ± SD (mg/dl)	236.21±79.1	226.1±68.34	198.2±45.72	0.005	0.58	197.03±54.18	191.41±61.08	187.27±54.49	0.21	0.17
Mean HbA1±SD mmol/mol(%)	63 (7.95±1.27)	58 (7.44±1.05)	58 (7.42±1.0)	0.014	0.46	60 7.6±1.17	58 7.47±0.95	59 7.52±0.98	0.14	0.07
Diabetes medications OHD Insulin+ OHD	34 04	34 04	34 04			34 03	34 03	34 03		

Cerebral haemodynamics

Longitudinal changes in various cerebral hemodynamic parameters showed similar trends in both MCAs. In summary, all TCD parameters except RI showed significant change at day 90 in the yoga intervention group in the RM-ANOVA analysis of ITT as well as PPA data. The control group did not show any significant changes.

**Figure 2:**
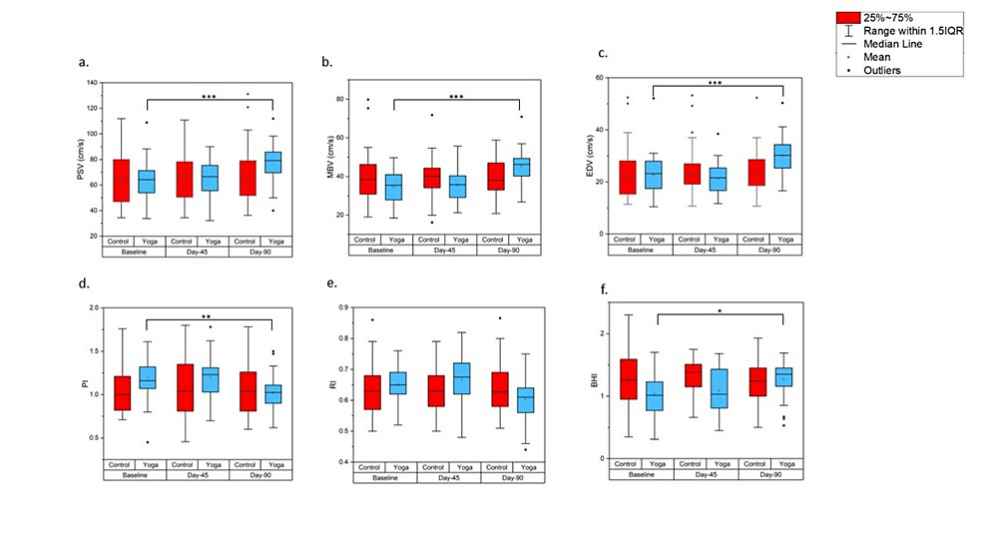
Boxplots of changes in the cerebral haemodynamics before and after three months of yoga intervention (a) PSV: peak systolic velocity; (b) MBV: mean blood flow velocity; (c) EDV: end-diastolic velocity; (d) PI: pulsatility index; (e) RI: resistance index; (f) BHI: breath holding index; *p<0.05, **p<0.01, ***p<0.001.

ITT analysis of the MBV showed a significant within-subject effect in the yoga group (F(2,146) = 18.03, p<0.001). A pairwise comparison of the yoga intervention group showed a significant improvement at day 90 (mean difference −10.85%, 95% CI (−13.26, −6.15), p<0.001) when compared to baseline. Similarly, the PI of the yoga group also showed significant improvement on day 90 (mean difference 0.04%, 95% CI (0.011, 0.074), p=0.005). Changes in the cerebral haemodynamics are shown in Figure 2.

Between subject effect of all the TCD parameters was not significant except for the BHI, which showed significant improvement (F (1,73) = 5.31, p=0.02). BHI also showed improvement in within-subject analysis (F (1.79, 130.99) = 3.84, p=0.02). A pairwise comparison of the yoga group showed significant improvement on day 90 (mean difference -0.23%, 95%CI (-0.43, -0.03), p=0.02). ITT analysis and PPA of cerebral haemodynamics are shown in Tables 4-5, respectively.

**Table 4:** Intention to treat analysis of middle cerebral artery BHI: breath holding index, EDV: end diastolic velocity in cm/s, MBV: mean blood flow velocity in cm/s, PI: pulsatility index, PSV: peak systolic velocity in cm/s, RI: resistance index, SD: standard deviation; *p<0.05, **p<0.01, ***p<0.001.

	Yoga group (n=38) within group					Control group (n=37) within group					Between group P-value
Variable	Baseline	Day 45	Day 90	p value (day 45)	p-value (day 90)	Baseline	Day 45	Day 90	p value (day 45)	p-value (day 90)	
Mean PSV±SD	63.5±15.9	64.9±13.7	76.1±18.5	1.00	<0.001***	64.6±20.5	66.8±18.3	67.7±21.3	0.79	0.42	0.63
Mean MBV±SD	34.5±8.7	35.6±7.2	45.4±8.5	1.00	<0.001***	40.2±13.3	38.7±11.1	39.5±10.1	0.92	1.00	0.63
Mean EDV±SD	22.6±7.7	21.3±5.6	30.1±6.7	0.78	<0.001***	23.5±10.1	24.3±9.1	24.4±8.0	1.00	1.00	0.72
Mean PI±SD	1.2±0.3	1.2±0.3	1.0±0.2	1.00	0.001^**^	1.1±0.3	1.2±0.6	1.1±0.3	0.42	0.37	0.55
Mean RI±SD	0.6±0.1	0.7±0.1	0.6±0.2	0.52	0.13	0.6±0.1	0.6±0.1	0.6±0.1	1.00	1.00	0.91
Mean BHI ±SD	1.0±0.3	1.1±0.3	1.3±0.3	1.00	0.019^*^	1.2±0.4	1.3±0.3	1.3±0.5	0.45	1.00	0.02^*^

**Table 5:** Per protocol analysis of transcranial doppler findings of middle cerebral artery BHI: breath-holding index, EDV: end-diastolic velocity in cm/s, MBV: mean blood flow velocity in cm/s, PI: pulsatility index, PSV: peak systolic velocity in cm/s, RI: resistance index, SD: standard deviation; *p<0.05, **p<0.01, ***p<0.001.

	Yoga group (n=29) within group					Control group (n=29) within group					Between group P-value
Variable	Baseline	Day 45	Day 90	p value (day 45)	p-value 90)	Baseline	Day 45	Day 90	p value (day 45)	p-value (day 90)	
Mean PSV±SD	61.6±15.2	63.7±14.3	76.2±13.4	1.00	<0.001***	64.4±18.9	66.6±16.6	65.6±15.0	1.00	1.00	0.66
Mean MBV±SD	34.5±8.6	35.5±7.4	45.1±7.9	1.00	<0.001^***^	40.2±12.8	39.8±11.7	38.8±9.9	1.00	1.00	0.61
Mean EDV±SD	20.9±6.2	21.4±5.6	29.6±6.5	1.00	<0.001^***^	23.9±9.6	24.4±8.9	23.7±6.9	1.00	1.00	0.97
Mean PI±SD	1.2±0.2	1.2±0.3	1.0±0.2	1.00	0.002^**^	1.0±0.3	1.1±0.3	1.1±0.3	0.84	0.37	0.26
Mean RI±SD	0.6±0.1	0.7±0.1	0.6±0.7	1.00	0.005^**^	0.6±0.1	0.6±0.1	0.6±0.8	1.00	1.00	0.62
Mean BHI ±SD	1.0±0.3	1.1±0.3	1.3±0.3	0.91	0.027^*^	1.3±0.4	1.3±0.4	1.3±0.3	0.75	1.00	0.015^*^

Cognitive assessment

Longitudinal assessments of the cognitive performance of ITT analysis and PPA are presented in Tables 6-7, respectively. ITT analysis showed a significant within-subject effect in the score of the forward Corsi block test (F (1.81,132.39) =10.83, p<0.001). Pairwise comparison of the yoga intervention group showed significant improvement at day 45 (mean difference −4.8%, 95% CI (−8.83, −0.82), p=0.013) and day 90 (mean difference −12.13%, 95% CI (−17.48, −6.78), p≤0.001) assessments, when compared to the assessments at recruitment. No significant changes were noted in the control group.

**Table 6:** Intention to treat analysis of Corsi block tapping test F_Span: Forward Corsi-block span, F_Score: Forward Corsi-total score, B_Span: Backward Corsi-block span, B_Score: Backward Corsi - total score, SD: standard deviation, *p<0.05, **p<0.01, ***p<0.001.

	Yoga group (n=38) within group					Control group (n=37) within group					Between group P-value
Variable	Baseline	Day 45	Day 90	p value (day 45)	p-value (day 90)	Baseline	Day 45	Day 90	p value (day 45)	p-value (day 90)	
Mean_F_Span±SD	5.44±0.76	5.66±0.84	6.01±0.70	0.10	<0.001^***^	5.62±0.72	5.56±0.63	5.46±0.63	1.00	0.65	0.24
Mean_F_ Score±SD	44.00±13.49	48.83±14.57	56.12±14.25	0.13	<0.001^***^	46.97±11.46	46.88±9.65	47.65±11.5	1.00	1.00	0.31
Mean_B_ Span±SD	5.60±0.75	5.84±0.63	6.06±0.69	0.06	<0.001^***^	5.64±0.67	5.50±0.49	5.71±0.65	0.47	1.00	0.09
Mean_B_ Score±SD	46.55±12.94	51.72±11.87	57.08±13.75	0.008^**^	<0.001^***^	48.21±11.91	46.92±10.05	49.44±11.17	1.00	1.00	0.15

**Table 7:** Per protocol analysis of Corsi block tapping test F_Span: Forward Corsi-block span, F_Score: Forward Corsi-total score, B_Span: Backward Corsi-block span, B_Score: Backward Corsi-total score, SD: standard deviation, *p<0.05, **p<0.01, ***p<0.001.

	Yoga group (n=29) within group					Control group (n=29) within group					Between group P-value
Variable	Baseline	Day 45	Day 90	p-value (day 45)	p-value (day 90)	Baseline	Day 45	Day 90	p value (day 45)	p-value (day 90)	
Mean_F_ Span±SD	5.51±0.73	5.72±0.88	6.10±0.72	0.32	<0.001^***^	5.62±0.72	5.58±0.62	5.41±0.62	1.00	0.39	0.12
Mean_F_ Score±SD	44.89±12.89	49.65±15.18	57.44±14.78	0.07	<0.001^***^	47.03±12.89	47.13±10.04	46.86±11.98	1.00	1.00	0.19
Mean_B_ Span±SD	5.55±0.78	5.86±0.69	6.06±0.75	0.02^*^	<0.001^***^	5.62±0.62	5.48±0.50	5.72±0.64	0.71	1.00	0.14
Mean_B_ Score±SD	45.93±13.59	52.00±12.89	57.34±14.86	0.008^**^	<0.001^***^	47.72±11.21	46.51±10.38	49.00±12.16	1.00	1.00	0.17

There was a significant within-subject effect in the score of the backward Corsi block test (F (2,146) = 13.72, p<0.001). Pairwise comparison of the yoga intervention group showed a significant improvement at day 45 (mean difference −5.17%, 95% CI (−9.23, −1.1), p=0.008) and day 90 (mean difference −10.52%, 95% CI (−14.74, −6.31), p≤0.001) assessments, when compared to the baseline. Similarly, the within-subject effect in the span of the backward Corsi block test showed a significant result (F (2,146) = 7.910, p<0.001). A pairwise comparison of the yoga intervention group showed a significant improvement at day 90 (mean difference −0.46%, 95% CI (−0.72, −0.20) p<0.001). The PPA of both forward and backward Corsi block tests showed similar results as the ITT analysis.

Correlation test

Table 8 shows Pearson’s correlation test. The present study did not show any significant correlation between cerebral haemodynamics and cognition.

**Table 8:** Pearson’s correlation test r^2^: Pearson's correlation coefficient.

Variable	MBV	
	r^2^	p-value
Corsi_Forward_Score	0.124	0.457
Corsi_Backward_Score	−0.054	0.745

## Discussion

Our study shows that yoga intervention for 90 days was associated with the improvement of cerebral hemodynamic parameters and the cognitive domain of visio-spatial working memory among patients with T2DM. Several factors are believed to be responsible for the improvement in cerebral hemodynamic parameters in subjects practicing yoga. Yoga is known to reduce stress by reducing the activity of the HPA axis and by activating the parasympathetic nervous system [21]. Yoga practices are known to cause vasodilation, which improves the nutrition flow towards the brain [22]. Also, yoga improves vascular endothelial function [23]. The inverted postures of the asana are known to improve blood circulation towards the head region.

T2DM is associated with impaired CVR, reduced blood flow velocities and increased PI and RI [24]. A previous study evaluated the effect of aerobic exercise in T2DM patients and showed improvement in the CVR, especially among the elderly population [25]. Our findings are in agreement with these observations. Yogic breathing techniques are known to improve cerebral haemodynamics. Kumbhaka (holding the breath after deep inhalation) is known to increase blood flow velocity and decrease PI [16]. Right nostril yogic breathing improves oxygenation and blood volume towards the prefrontal cortex [26]. Another study assessing the effect of meditation on cerebral blood flow using functional near-infrared spectroscopy (fNIRS) showed improved cerebral oxygenation as well as cognitive performance [27].

Our findings on the improvement of visuospatial working memory related to the cognitive domain are in agreement with the previous studies on yoga and cognition. Yoga is known to improve cognition in T2DM patients, supported by the electrophysiological evidence of an improvement in the latency and amplitude of P300 [10].

Yoga practice is believed to improve cognitive skills among healthy subjects as well as in those with neurological disorders, attributed to changes in autonomic function, structural changes in the brain, metabolic enhancement, and improved brain network connectivity [28].

The improvement in cognition can be attributed to the neuroprotective effect of yoga, which demonstrates a positive effect of yoga practice on the structure and function of the hippocampus, amygdala, prefrontal cortex, cingulate cortex, and brain networks [29].

The limitation of the study is the small sample size and relatively shorter duration of the intervention. Furthermore, whether the improvements in cerebral hemodynamics and cognition in the yoga intervention group are sustained for longer periods remains unknown. Additionally, despite the improvements in cerebral hemodynamics, we did not find any significant correlation between cerebral haemodynamics and cognition. Perhaps a smaller sample size and shorter study duration accounted for this observation and future larger and longer studies may provide better answers.

## Conclusions

The three-month yoga intervention was associated with an improvement in cerebral haemodynamics. The study also revealed an improvement in visio-spatial working memory among patients with T2DM. The study did not show any correlation between the improvement in cerebral haemodynamics and working memory. We recommend larger and longer studies on yoga intervention for T2DM patients to evaluate whether such benefits are sustained and improve their quality of life.
